# Insecticide‐treated nets distribution campaign: Physical integrity, usage and sustainable disposal of end‐of‐life insecticide‐treated nets under operational settings in Odisha, India

**DOI:** 10.1111/tmi.14107

**Published:** 2025-04-21

**Authors:** A. N. Shriram, Mustafa Baig, D. K. Panigrahi, B. Vijayakumar, S. S. Sahu, Tatvadarshi Dash, Ashwani Kumar

**Affiliations:** ^1^ ICMR‐Vector Control Research Centre Puducherry India; ^2^ ICMR – Vector Control Research Centre, Field Unit Koraput Odisha India; ^3^ Community Health Centre Kalahandi Odisha India

**Keywords:** disposal, end‐of‐life ITNs, malaria, Odisha, physical status, practices

## Abstract

**Objectives:**

Prior to 2017, Odisha accounted for 50% of all *Plasmodium falciparum* cases in India. The ‘National Strategic Plan’ for malaria elimination had distributed 11.3 million insecticidal treated nets (ITNs) to 23 million individuals in Odisha's 17 malaria‐endemic districts by 2017. In 2021, the National Centre for Vector‐Borne Disease Control replaced end‐of‐life ITNs. India needs official regulations on end‐of‐life ITNs collection and disposal mechanisms. A pilot study was undertaken to understand community practices and perceptions on end‐of‐life ITNs.

**Methods:**

The 2021 study was conducted during mass ITN replenishment in a campaign mode at Koksara Community Health Centre in Odisha's Kalahandi region. ITN conditions were evaluated using structured questionnaires and household interviews. End‐of‐life ITNs were evaluated for chemical analyses. Door‐to‐door assessments of net conditions included noting their presence, attrition rates and fabric integrity. Officials from the Department of Health were consulted on eco‐friendly disposal techniques.

**Results:**

In the study region, 6022 ITNs were distributed, of which 5879 (97.6%) were available and 143 (2.4%) were reported as missing 43 months after the campaign (2017). One net per 2.1 persons was distributed. Of the 5879 ITNs, 84.2% were torn, 931 (15.8%) were in good condition and 3472 (59.1%) were serviceable. When combining the ITNs in good condition and those that were serviceable, 74.9% were deemed usable. A total of 3050 respondents were interviewed. Most respondents (98%, 2935/3050) were willing to exchange old ITNs for new ones (92.5%, 5437/5879) when replaced with new ones, highlighting the need for a disposal mechanism at both programme and household levels. Additionally, 61.6% of respondents chose to keep their ITNs, while others repurposed them (7.3% for covering items, 3.0% for fencing, 4.6% for nursery saplings, 5.6% for fishing, 0.4% for other uses). The end‐of‐life PermaNet 2.0 nets contained an average of 0.33 ± 0.35 g/kg (15.3 mg/m^2^), while the new nets contained an average of 1.4 g/kg ± 25% (55 mg/m^2^).

**Conclusions:**

Community input on end‐of‐life ITN disposal contributes to the development of evidence‐based decision support materials, facilitating the formulation of a strategy for the systematic collection and safe disposal of used nets. The lack of an operationally viable solution for the secure disposal of end‐of‐life ITNs within the National Programs underscores the urgent need for a comprehensive policy framework.

## INTRODUCTION

Long‐lasting insecticidal treated nets (ITNs) are an integral part of malaria prevention and control strategies. In order to eliminate malaria worldwide by 2030, approximately 2900 million (2.9 billion) ITNs have been distributed to malaria‐endemic nations (World Malaria Report, 2023). The widespread distribution of ITNs in India conforms to a globally recognised and effective malaria control strategy [1, 2, 3]. India's usage of ITNs has increased significantly in recent years. The World Health Organization (WHO) approved universal coverage target (one net for every two persons) is being implemented, and ITN use is prevalent in many endemic countries [4, 5, 6].

ITNs inhibit man‐vector contact [7] by creating both physical and insecticidal barriers. These barriers should retain their effectiveness despite repeated washing (up to 20 times) and throughout extended use. ITNs, like any other product, have a shelf life; however, they do not last forever. Comparing ITNs to conventional, untreated or manually treated nets reveals that ITNs provide protection for a significantly extended duration. Their quality and amount of insecticide diminish with use and time. According to WHO recommendations for public health use [8, 9], ITNs must be replaced to maintain their effectiveness in the prevention and control of malaria. Despite their long lifespan, ITNs must be replaced to maintain their effectiveness in the prevention and control of malaria. Both new and old nets have been used to create fishing nets, dryers and wedding dress fabric [10]. African media [11, 12] and scholarly articles [13] have discussed the use of ITNs for purposes other than preventing malaria.

The primary concern is the lack of scientific research in India on the misuse of ITNs. Could the reported misuse instead be characterised as repurposing, particularly when ITNs have reached the end‐of‐life?

It is possible to differentiate between using an ITNs for something other than its intended use and misusing it. Repurposing can be beneficial (act as a mosquito barrier) or neutral (offer no mosquito barrier). Misuse [14] includes the use of a new ITN, the use of an ITN that contaminates the ecosystem with insecticides and the use of a new ITN for fishing.

Contrary to prevalent belief, prior research indicates that misuse is uncommon. According to Eisele et al.,[12] there is scant evidence to support claims of widespread misuse. Only 3% of households in regions where fishing is a significant source of income used ITNs for fishing [12]. Only 5.3% of households in Sierra Leone self‐reported using bed nets for purposes other than mosquito prevention [13]. In order to understand the anticipated components and outcomes of each phenomenon in the context of malaria prevention and control, it is necessary to differentiate between misuse and repurposing. According to data [14, 15], ITNs are repurposed when households believe they are torn or at the end of life.

Odisha State, India has had high rates of malaria for decades, accounting for approximately 40% of the nation's total malaria burden and 50% of all *Plasmodium falciparum* cases documented until 2017 (Source: National Centre for Vector Borne Diseases Control, NCVBDC, Odisha). ITN universal coverage and ‘*Durgama Anchalare Malaria Nirakaran*’ (elimination of malaria in inaccessible regions) were highlighted in the two new interventions introduced in mid‐2017 in 17 high malaria endemic districts of Odisha State under the National Framework of Malaria Elimination (NFME) and National Strategic Plan (NSP) [16, 17, 18]. In 2017, 11.3 million ITNs were distributed to protect 23 million people in 17 districts of Odisha with high *P. falciparum* endemicity. As a consequence, it is believed that ITN coverage is reasonably high, moving closer to the universal coverage goal of one net for every 2.5 at‐risk population members, as recommended by National standards. According to the WHO guidelines, the efficacy criteria requires that ≥80% of the ITNs in routine household use should meet the efficacy cut‐off criteria in cone bioassays, that is, mortality of ≥80% and/or mosquito knockdown of ≥95%; and in case of failed ITNs ≥90% blood feeding in tunnel tests [19]. Premature bio‐efficacy loss of ITNs remains a concern [20]. Given the decreased fabric integrity, this raises the question of whether the bio‐efficacy of ITNs is sufficient to rely on the insecticidal effect as a balancing mechanism.

After 2 years of household use of PermaNet® 2.0 ITNs in rural Uganda, bio‐efficacy testing revealed 74% functional mortality [21]. Similarly, after 2 years of use in Ethiopia, Permanet® 2.0 ITNs demonstrated 67%–72% mortality against *Anopheles arabiensis* [22]. Only 73% of ITNs evaluated in Cambodia met the WHO requirements for use against *Anopheles dirus* (s.s.) susceptible strains [23]. However, in hilltop, foothill (FH) and plain (PL) villages in Koraput district, Odisha, 94%, 80% and 63.8% of Permanet® 2.0 ITNs were physically present. ITNs met the requirements for bio‐efficacy [24].

India and other nations must establish their own protocols for the collection and secure disposal of end‐of‐life ITNs, as no WHO guidelines are currently available for the purpose. ITN distribution programs are supported by ITN durability monitoring, which guides the timing of ITN distribution campaigns to accomplish a sustainable impact. This study examines the physical integrity, residual insecticide content, community usage practices and attitudes towards the disposal of end‐of‐life ITNs, 43 months after the initial distribution campaign in 2017 across 17 districts of Odisha. In addition, we investigate the attitudes of health authorities who are directly involved in the management of vector‐borne infections (VBDs) and those who are not regarding end‐of‐life ITNs, as well as their knowledge and comprehension of malaria control strategies and mosquito‐borne infections.

## METHODS

### Study area

**FIGURE 1:**
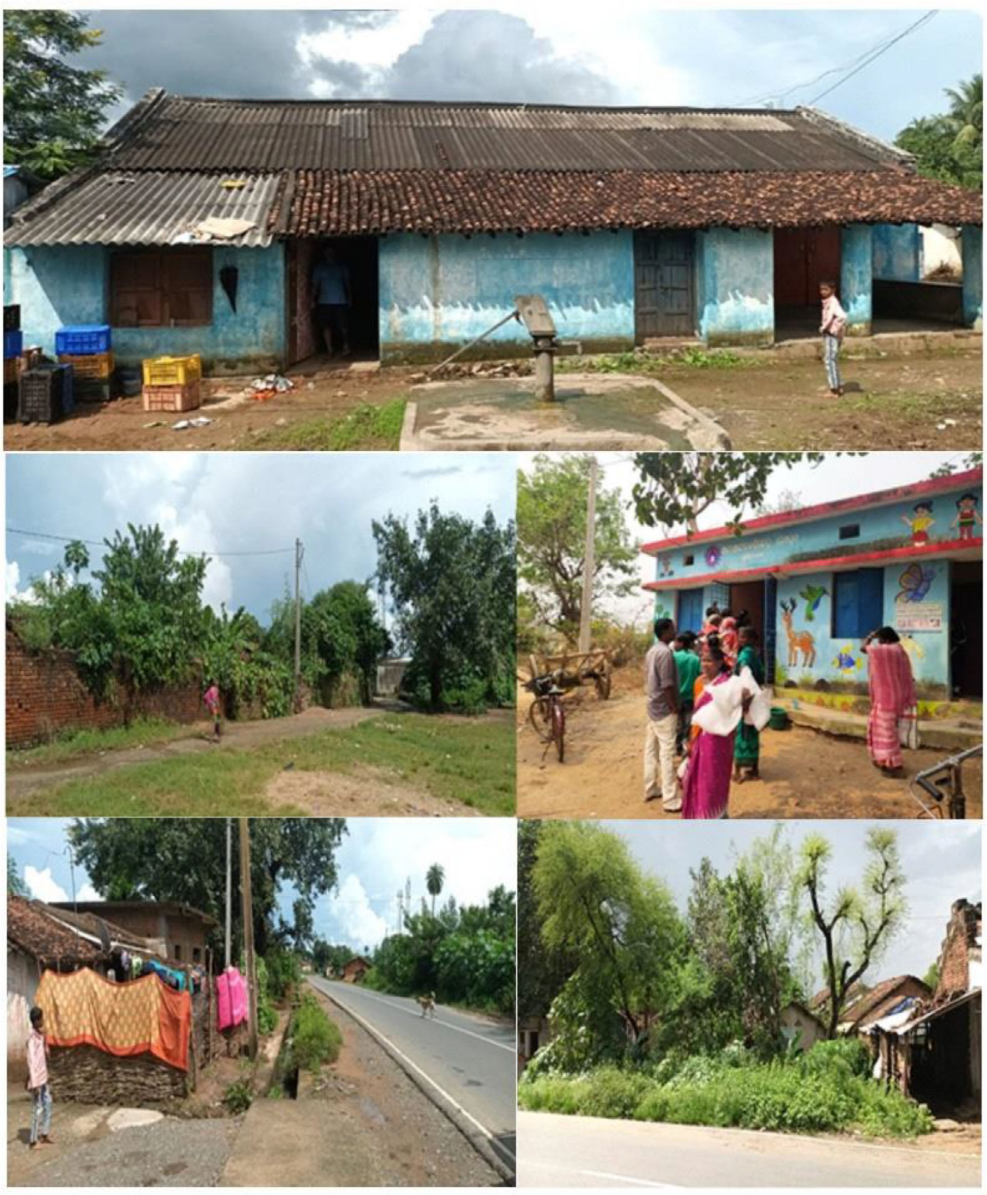
Picture showing terrain and housing pattern in Koksara, Kalahandi District, Odisha.

The investigation was conducted in March, 2021, at the same time when the Koksara Community Health Centre (CHC) in the Kalahandi region of Odisha State was mass replenishing its supply of new ITNs (Figure 1). As of 2017, the district had 1,695,538 residents. In July and August of 2017 [16, 17, 18], the state NCVBDC initially distributed ITNs. The district of Kalahandi, which is situated in the southwest part of the Indian state of Odisha, lies between 19.3′ N and 21.5′ N latitude and 82.20′ E and 83.47′ E longitude. The district occupies an area out of 8365 km^2^, making it the seventh largest of Odisha's 30 districts in terms of size. The geography of Kalahandi consists of plains, hills and mountains. Its boundary with the districts of Nabarangpur, Koraput, Rayagada and Kandhamal is rugged and hilly. Over one‐third of the district consists of dense forest, while agriculture is the predominant land use. The Annual Parasite Incidence (API) for the district ranged from 18.97 in 2009 to 2.1 in 2022, with no malaria‐related deaths reported during this period (2009–2022) (Source: Office of the Chief District Medical Officer, Koraput). *P. falciparum* is the most prevalent form of malaria in the region. The region's anopheline fauna is abundant. The two primary malaria vectors in the area are *Anopheles fluviatilis* and *Anopheles culicifacies* [25].

**FIGURE 2:**
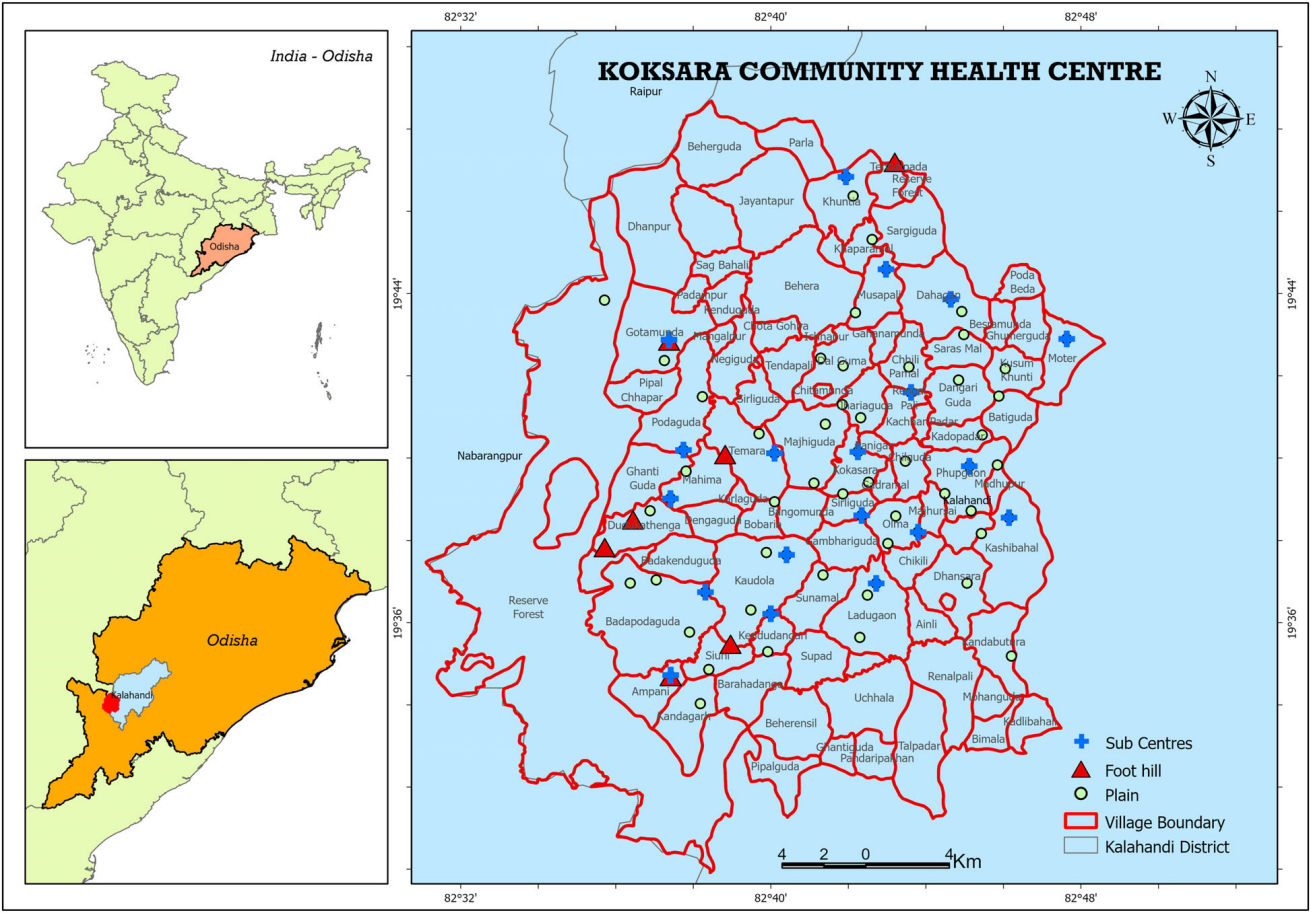
Map of Koksara Community Health Centre, Kalahandi District, Odisha showing the study villages.

There are approximately 128,257 residents in Koksara CHC, who are dispersed across 130 villages and are predominantly Banjara (70%). The CHC consists of 19 sub‐centres. Figure 2 shows that both urban and rural study villages were selected. The urban area (5%) is notable for its high population density, numerous commercial establishments and residential homes with little vegetation. In this region, electricity, paved roads and piped water are all fairly abundant. The vast majority of tribal people (95% of them) reside in villages with mud and tiled houses and extensive agricultural lands. The rural area is distinguished by its minimal population density. The CHC's API ranged from 5.81 in 2013 to 0.42 in 2022, and there was no malaria‐related mortality (Source: Office of the Chief District Medical Officer, Kalahandi).

### Sample size

The sample size of 1537 was calculated with a 2% accuracy error, an estimated 80% retrieval rate of ITNs and a 95% confidence interval. Taking into account the presumed two‐fold impact of the design, the total sample size was 3073. This calculation determined that sampling would be conducted in a total of 57 villages and four urban areas, each with 50 fixed households. Consequently, for the purpose of this research, three villages from each of the 19 sub‐centres were selected at random and additionally one urban area was included in each of the following sub‐centres, viz. Ampani, Koksara, Ladugaon and Phupugaon. Thus, there were 61 study sites included in the survey.

Sixty‐one study sites were selected at random to reflect the urban and rural environments, respectively, with the assistance of local officials and public health personnel. To determine how many ITNs were distributed to each household, the selected villages were tallied. The survey was conducted in collaboration by the Indian Council of Medical Research‐Vector Control Research Centre (ICMR‐VCRC) and NCVBDC at Koksara CHC.

### Implementation of the study

The head of each household (an adult man or woman who was available) in the study villages was interviewed using pre‐tested structured questionnaires to determine the physical condition of ITNs at the time of retrieval: (a) unusable torn nets, (b) torn nets but in usable condition, (c) nets in good condition, and (d) to understand the customs of the community regarding the use of old nets; and (e) A unique number was assigned to each end‐of‐life ITN during the survey in order to track ITN attrition, availability and condition.

### Assessment of the age of surviving/torn nets

During the household surveys, participants were asked about the origin of their nets. The questions included the year of receipt, the source of the net and additional details to ascertain if the nets were obtained through official distribution campaigns. Furthermore, the ITNs were labelled with ‘CIB Registered, NVBDCP Supply, not for sale’, along with the logos of the National Health Mission and NVBDCP. NCVBDC's historical records were accessed for information on the final ITN distribution dates in the designated locations. In India, ITNs are distributed exclusively by the NCVBDC and are not available through other sources or commercial markets.

### Community consent

Prior to the project's launch, the village chiefs were contacted and apprised of the project's specifics and objectives. The Accredited Social Health Activists (ASHAs) were then briefed on the initiative in group meetings, and their assistance was in raising awareness among the local populace within their sphere of influence. To aid in the interviewing of female candidates, the interview team included female employees.

### Chemical assay method

While conducting chemical testing, the NCVBDC, Odisha, replenished end‐of‐life ITNs with brand new ITNs. From 57 villages, 30 villages were randomly selected. Of these randomly selected villages, one net was taken from the randomly selected household for the purpose of analysing chemical residue. In accordance with WHO standards from 2013, sample meshes were used for the chemical analysis. Positions 2, 3, 4 and 5 from each of the 30 selected nets were then cut with sharp scissors into four rectangular portions measuring 30 cm by 30 cm. As the netting fabric in position 1 is routinely handled while being hidden under the bed or mattress, it is subjected to significant abrasion during normal use; therefore, no sample was taken from this position. Before being shipped to the Phytopharmacy Department at the Agricultural Research Centre in Gembloux, Belgium (a WHO Collaborating Centre), the sub‐samples were rolled and stored at +4°C in clean, fresh and labelled aluminium foil. In the laboratory facility, the four subsamples from each net were combined into one sample for chemical analysis.

### Assessment of the physical status of collected nets

In order to compute the attrition rate, 50 HHs were selected using systematic random sampling from each of the selected villages to conduct a survey and record the physical presence/absence and fabric integrity (hole index) of all the available nets.

### Generation of information for drawing guidelines

During the implementation of the project, meetings were conducted with relevant officials and authorities in an effort to investigate eco‐friendly options for the disposal of used ITNs and the availability of facilities and options in the area. The purpose of the consultations was to collect the opinions and experiences of respondents.

The participants were selected using a technique of purposive sampling from a variety of categories. This allowed both health officers involved in VBDs and those who were not to participate. Twenty health officers, including the Additional District Medical Officer (ADMO), Auxiliary Male Nurses (ANMs), Chief District Medical Officer (CDMO), Lady Health Visitors (LHV) and Multipurpose Health Supervisor (MPHS), were not involved in VBDs (Table 6); however, 27 health officers, including the Accredited Social Health Activist (ASHA), Anganwadi worker (AWW), Health Inspector, Health worker, Laboratory Technician (LT), Multi‐tasking staff (MTS), Medical officer and VBD Consultant, were involved in the study (Table 7).

A semi‐structured questionnaire was used to collect data regarding knowledge of mosquito‐borne diseases, mosquito breeding grounds, the current malaria situation compared with the previous year, malaria control measures, the lifespan of ITNs and disposal recommendations for end‐of‐life ITNs.

### Data analysis

Before being entered into an Excel spreadsheet, all data were captured using a data capture format. The data were presented as frequency percentages. A probability value of 0.05 or less was determined to be statistically significant. The Pearson correlation coefficient was used to assess the relationship between the insecticide concentration (g/kg) in the end‐of‐life ITNs and the mean mortality observed in cone bioassays. The difference in mean mortalities from cone bioassays was evaluated using a *t*‐test. STATA 14.2 (Texas, USA) was utilised for all statistical analyses.

## RESULTS

### Availability and physical integrity of ITNs

The survey covered 12,489 population across 61 villages located in the foothills (FH) and plains (PL) served by 19 sub‐centres. This included 3050 households, with one individual sampled per household, totalling 3050 individuals. The NCVBDC distributed a total of 6022 ITNs to the 3050 households in the study area (Table 1). During the distribution campaign, an average of 2.1 individuals received an ITN. It was determined that 5879 (97.6%) of these ITNs were available, while 143 (2.4%) were missing. For the community surveys, 5879 ITNs (FH: 566; PL: 5313) were physically inspected from 3050 households. Of the 5879 ITNs, holes were found in 297 (52.5%) nets in foothill villages and 2441 (45.9%) in plains villages (Table 2). The total number of holes detected in ITNs of sizes 1, 2, 3 and 4 in the 61 villages is provided in Table 3 [29, 30]. The average hole index of ITNs in FH and PL villages was 859.08 and 564.9, respectively. According to the pHI score, 931 (15.8%) ITNs were in ‘good’ condition (pHI 64), 3472 (59.1%) ITNs were in serviceable condition (pHI 642) and 1476 (25.1%) ITNS were in replaceable condition (pHI > 642) due to excessive tornness (Table 4). Holes were found in 297 (52.5%) and 2441 (45.9%) of the 5879 ITNs (Table 2). The total number of holes detected in ITNs of sizes 1, 2, 3 and 4 in the 61 villages is provided in Table 3. The average hole index of ITNs in FH and PL villages was 859.08 and 564.9, respectively. According to the pHI score, 931 (15.8%) ITNs were in ‘good’ condition (pHI 64), 3472 (59.1%) ITNs were in serviceable condition (pHI 642), and 1476 (25.1%) ITNS were in replaceable condition (pHI > 642) due to excessive tornness (Table 4). PL villages had a statistically significant difference (1614.8; *p*‐value 0.00001) greater proportion of ITNs in acceptable condition (16.9%) compared with FH villages (5.6%).

**TABLE 1:** General information on the distribution of long‐lasting insecticidal nets in study villages of Koksara CHC, Kalahandi District, Odisha.

General information	Terrain		Total
Terrain	Foothill	Plain	
Total population of surveyed village	7177	52,707	59,884
Total number of HH	1644	12,450	14,094
Number of HH selected	300	2750	3050
Total population in HHs surveyed	1162	11,327	12,489
No. of ITNs provided to surveyed HHs	574	5448	6022
No. of ITNs inspected	566	5313	5879

Abbreviations: ITNs, insecticidal treated nets; HHs, households.

**TABLE 2:** Distribution of holes in long‐lasting insecticidal nets inspected in study villages of Koksara, CHC, Kalahandi District, Odisha.

Village	No. of ITNs surveyed	No. of ITNs with holes (%)
FH	566	297 (52.5%)
PL	5313	2441 (45.9%)

Abbreviations: FH, foothill; ITNs, insecticidal treated nets; PL, plain.

**TABLE 3:** Physical integrity of insecticidal treated nets in the study villages of Koksara, CHC, Kalahandi District, Odisha.

Hole size category	Hole size (cm)	Weightage of hole sizes (WHO)	FH (*n* = 566)		PL (*n* = 5313)	
			No. of holes	Hole index	No. of holes	Hole index
1	0.5–2.0	1	1469	1469	13,916	13,916
2	2–10	23	1298	29,854	11,813	271,699
3	10–25	196	702	137,592	4853	951,188
4	>25	578	549	317,322	3053	1,764,634
Mean hole index				**859.08**		**564.9**

Abbreviations: FH, foothill; PL, plain.

**TABLE 4:** Physical condition of distributed insecticidal treated nets in the study villages of Koksara, CHC, Kalahandi District, Odisha.

Conditions of nets (pHI)	FH (%) (*n* = 566)	PL (%) (*n* = 5313)	Total (%) (*n* = 5879)
Good (0–64 pHI)	32 (5.6)	899 (16.9)	931 (15.8)
Serviceable (65–642 pHI)	352 (62.2)	3120 (58.7)	3472 (59.1)
Too torn (>643 pHI)	182 (32.2)	1294 (24.4)	1476 (25.1)
Total	566 (100)	5313 (100)	5879 (100)

Abbreviations: FH, foot hill; PL, plain.

### Net survivorship and attrition

Totalling 3050 households, 6022 ITNs were distributed. After 43 months, 97.6% (*n* = 5879) of the ITNs were physically present, while 2.4% (*n* = 143) were missing. 92.5% (*n* = 5437) of the 5879 ITNs were retrievable. The net losses were caused by disposing of nets due to wear and tear 29 (0.48%), giving away 59 (0.97%), and using nets for other purposes 55 (0.91%). Compared with FH village 8, PL village 135 had a 2.5% greater attrition rate. During the survey, a total of 5313 (97.5%) and 566 (98.6%) individuals were physically present in the PL and FH villages, respectively (Table 5).

**TABLE 5:** Attrition and net‐survivorship at 43‐month post‐distribution of insecticidal treated nets (ITNs) in study villages.

Attrition	FH (%) (*n* = 574)	PL (%) (*n* = 5448)	Total (%) (*n* = 6022)
Attrition‐1 Wear and tear (disposed)	2 (0.34)	27 (0.49)	29 (0.48)
Attrition‐2 Sold/stolen/Gifted	6 (1)	53 (0.97)	59 (0.97)
Attrition‐3 Used for other purpose	0 (0.00)	55 (1.00)	55 (0.91)
Total attrition	8 (1.4)	135 (2.50)	143 (2.80)
ITNs present (survivorship)	566 (98.6)	5313 (97.50)	5879 (97.62)

Abbreviations: FH, foothill; PL, plain.

### Willingness of the community to return old ITNs, alternate uses of nets and opinion on disposal of end‐of‐life ITNs

**FIGURE 3:**
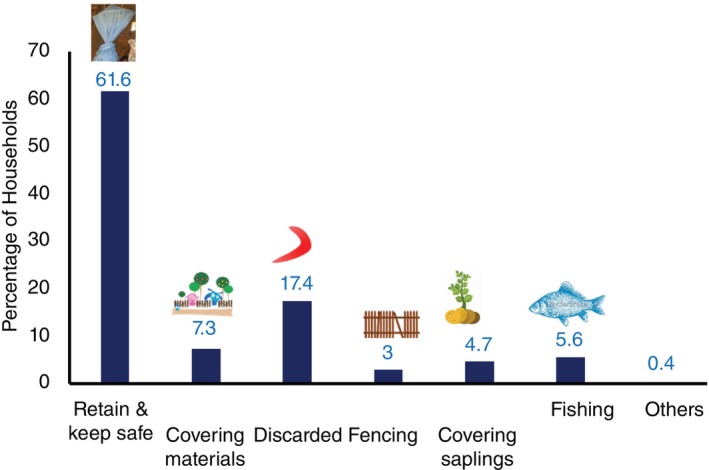
Uses of end‐of‐life insecticidal treated nets as observed in the study (images are royalty‐free images obtained from public domain in vectors).

In total, 2985 out of 3050 respondents across 61 study villages and 19 sub‐centres indicated that they would gladly surrender their old ITNs if they were replaced with new ones (92.5 percent, 5437 out of 5879). Of the 3050 respondents, 1879 (61.6%) wanted to retain the ITNs, 222 (7.3%) used them for covering objects/items like covering earthen pots, small mud pots used for tapping toddy and protecting hatched chickens from predators. Five hundred and thirty‐one (17.4%) ITNs were discarded after damage, 91 (3%) used the ITNs for fencing, 143 (4.7%) used the ITNs on saplings in the nursery, 172 (5.6%) used the ITNs for fishing, and 12 (0.4%) for other uses (Figure 3). Among the total respondents, 1255 (41.2%) expressed a preference to discard the ITNs, while 1202 (39.4%) preferred to store them safely. Additionally, 506 respondents (16.6%) were uncertain about disposal methods, 44 (1.4%) suggested burning the ITNs and 43 (1.4%) proposed burying them.

### Chemical analysis—Deltamethrin content in PermaNet 2.0

**FIGURE 4:**
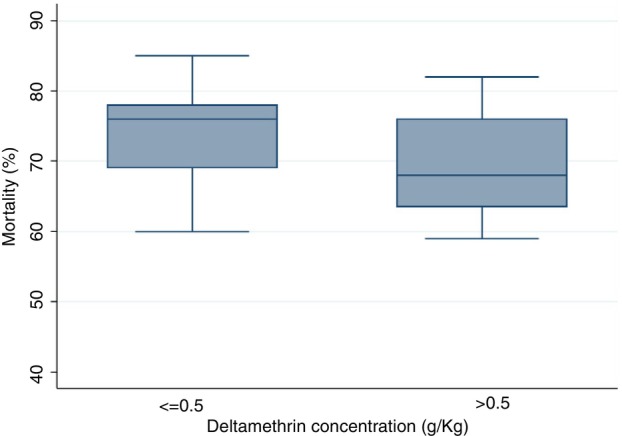
Bio‐efficacy of the end of life insecticidal treated nets and insecticidal content (*n* = 30 net samples).

According to WHO regulation 333/LN/1 (September 2020), the target dose and tolerance limit for the deltamethrin content in PermaNet 2.0 is 1.4 g/kg ± 25% (1.1–1.8 g/kg) for 100 and 150 denier yarn. Because the fabric weight was closer to 40 than 30 g/m^2^, it was assumed that the samples were 100 and/or 150 deniers. Thirty samples of end‐of‐life PermaNet 2.0 had a mean ± SD deltamethrin content of 0.33 ± 0.35 g/kg for 100 and/or 150 denier yarn at the end‐of‐life ITNs. This equates to 15.3 mg/m^2^, whereas the fresh nets contained 1.4 g/kg, corresponding to 55 mg/m^2^. The concentration of deltamethrin in the individual nets ranged from <0.01 g/kg to 1.26 g/kg, as shown in (Figure 4). The relative standard deviation (RSD) for the deltamethrin content on five distinct net sections taken from 30 end‐of‐life ITNs was 107.3%, showing heterogeneity of the distribution of the active ingredient within the nets.

### Knowledge and awareness of mosquito‐borne diseases, malaria control measures and end‐of‐life ITN among health officers, involved and not involved in VBDs

#### Health officers (not involved in VBDs)

One hundred percent of the 200 respondents were aware that mosquitoes can transmit disease. 55% (11 of 20) of these numerous respondents were aware that mosquitoes transmit dengue, filariasis, and malaria. Ten percent (2 of 20) of respondents were aware that mosquitoes can transmit dengue and malaria. Five percent (2 of 20) of respondents indicated that mosquitoes are capable of transmitting JE, dengue fever, and malaria. Only 5% of respondents were aware that in addition to malaria, mosquitoes can transmit dengue, filariasis, chikungunya, JE, and zika. However, all 20 respondents were aware that mosquitoes breed in bodies of water and transmit malaria. In addition, these 20 respondents reported a decline in malaria cases compared with earlier years.

Respondents were well‐informed regarding malaria prevention strategies. Fifty‐five percent of respondents were aware that ITNs, IRS and larval control are utilised to combat malaria. The remaining 45% of respondents indicated that the use of ITNs and IRS was an effective malaria control strategy.

The respondents had adequate direct knowledge of the ITNs' lifespan. 95% of respondents stated that the ITNs' lifespan was 3 years, while 5% believed it to be lengthier. 75% of respondents suggested returning ITNs to the state government for disposal once their useful lifetimes had expired. The remaining 25% believed that used ITNs should be returned to the State Government, while unused nets should be disposed of in a safe manner (Table 6).

**TABLE 6:** Responses of Health Officers (*n* = 20) not involved in VBDs with regard to the use and disposal of insecticidal treated nets (ITNs) distributed to the community in villages of Koksara CHC in Odisha.

S. No	Variable (questionnaires)	*n*	%
1	*Do you know that mosquito transmit diseases?*		
	Yes	20	100.0
2	*If yes, what are they?*		
	Malaria	1	5.0
	Malaria and dengue	2	10.0
	Malaria, dengue and JE	2	10.0
	Malaria, dengue and chikungunya	1	5.0
	Malaria, filariasis and dengue	11	55.0
	Malaria, filariasis, dengue and chikungunya	1	5.0
	Malaria, filariasis, dengue, chikungunya and JE	1	5.0
	Malaria, filariasis, dengue, chikungunya, zika and JE	1	5.0
3	*Do you know mosquitoes breed in water bodies?*		
	Yes	20	100.0
4	*Do you feel malaria has reduced compared to the previous years?*		
	Yes	20	100.0
5	*Could you tell what are the control measures taken for the control of malaria?*		
	Indoor Residual Spray/insecticide spray on the walls, long‐lasting ITNs/bed nets, Larval control (control of mosquito immature stages)	11	55.0
	Indoor Residual Spray/insecticide spray on the walls, Long‐lasting ITNs/bed nets	9	45.0
6	*Do you know the lifespan of a ITNs?*		
	3 years	19	95.0
	More than three years	1	5.0
7	*What are your suggestions for the disposal of old ITNs?*		
	Return back to the state Govt.	15	75.0
	Return back to the state Govt. and unused condition nets should be disposed of safely.	5	25.0

#### Health officers (involved in VBDs)

Malaria was cited by all 27 respondents (100%) as a health concern in the area. 26/27 (96.3%) participants were aware that mosquitoes transmit malaria. One in every 27 respondents, or 3.7%, believed that unsanitary conditions contributed to the spread of malaria.

Seventy‐eight percent of the 27 respondents were aware that IRS, ITNs and IEC are the primary intervention instruments for the management of malaria and mosquitoes. According to 14.8% of respondents, only ITNs are used to combat malaria and vermin. Very few respondents (3.7%) (1/27) indicated that IRS and IRS + ITNs were the intervention tools. These 27 respondents also believed that the incidence of malaria had decreased compared with previous years and that the at‐risk population consistently utilised ITNs.

Regarding the lifespan of ITNs, 12 (44.4%), 10 (37.0%), 3 (11.1%) of respondents, respectively, stated that it was 2, 3 and 1 year, and 1 (3.7%) each of the respondents believed that more than 5 years had passed and had a long lifespan, respectively. According to 24 (88.9%) of respondents, the expired ITNs may have some domestic use. Meanwhile, 1 (3.7%) of the respondents wished to return to the State Government and 2 (7.4%) wished to be burned (Table 7).

**TABLE 7:** Responses of Health Officers (*n* = 27) directly involved in vector borne diseases with regard to the use and disposal of old insecticidal treated nets (ITNs) in villages of Koksara CHC in Odisha.

S. No	Variable (questionnaires)	*n*	%
1	*Is malaria a health problem in your area?*		
	Yes	27	100.0
2	*If yes, could you tell what transmits malaria?*		
	Mosquito	26	96.3
	Unhygienic condition	1	3.7
3	*What are the intervention tools that are being used for controlling malaria and mosquito?*		
	IRS	1	3.7
	IRS and ITNs	1	3.7
	IRS, ITNs and IEC	21	77.8
	ITNs	4	14.8
4	*Now do you feel whether malaria has reduced compared to the previous years?*		
	Yes	27	100.0
5	*Do you feel that the community is using the ITNs regularly?*		
	Yes	27	100.0
6	*Do you know the life span of a ITNs?*		
	1 year	3	11.1
	2 years	12	44.4
	3 years	10	37.0
	5 years	1	3.7
	Long lifespan	1	3.7
7	*After the lifespan of the nets/or when it becomes old‐what is your opinion as to how the community will use them?*		
	Few are already returned back to the Govt.	1	3.7
	Use for domestic purpose	24	88.9
	Will burn	2	7.4
8	*Could you suggest some methods for safe disposal of ITNs?*		
	All nets to be handed over to Govt. org.	18	66.7
	New one to be used and burn the old ones	3	11.1
	New one to be used and keep the old ones at home	4	14.8
	The old nets again to be impregnated for use	2	7.4

## DISCUSSION

National Malaria Control Programs in numerous endemic nations rely heavily on the distribution of ITNs as a vector control strategy to prevent the spread of malaria. To ensure prompt replacement of ITNs, it is necessary to monitor the longevity, physical integrity and bio‐efficacy of the nets [26, 28]. Previous research [27, 30, 31, 32] has evaluated the efficacy of various ITN products in diverse eco‐epidemiological and socio‐cultural contexts. This is one of the few studies to examine the accessibility, physical integrity, durability, attrition and insecticidal content of ITNs in Odisha, India, while they were still in use. Additionally, the community's willingness to dispose of end‐of‐life ITNs, desire to return end‐of‐life ITNs, and views on the use of alternative nets were evaluated.

The ITNs are designed with a 3‐year life span. In the study area, the initial ITN distribution campaign took place in July 2017, meaning the subsequent campaign ideally should have occurred in June–July 2020. However, it was implemented approximately 7 months later in 2021. This slight extension beyond the standard 3‐year period offers valuable insights into the functional durability and bio‐efficacy of ITNs following typical use under field conditions. Such data allow us to assess whether ITNs continue to provide protective efficacy beyond the expected 3‐year period. In accordance with the recommendations of the NCVBDC (2017–2021), multiplying the number of dwellings by 2 and dividing by the total population multiplied by 2.5 will approximate the number of ITNs required for a given village. There are three distinct sizes of nets based on the number of individuals they can accommodate: size 1—180 × 100 × 150 cm (one net per individual); size 2—180 × 130 × 150 cm (one net per 1.8 individuals); whereas size 3—180 × 160 × 150 cm (one net per 2.5 individuals) [29]. This suggests that 6100 nets should have been supplied to 3050 families, but instead, 6022 nets were distributed, indicating that 98.7% of the households had been covered. In this study, we found that one net was given to an average of 2.1 people throughout the distribution campaign, and that 97.6% of the ITNs were still in use by the families 43 months following distribution. Other places include Odisha, where it was found that after 30 months of distribution, one net was given to an average of 2.3 people, 25% of the nets were not available, and over 42% of the population was not covered by the ITNs [24]. The results of this study show that >80% of the population (98.7%) had access to ITNs, proving that the access gap in this malaria‐endemic setting, which is aimed for universal coverage, is hardly noticeable. The WHO‐recommended level of 80% (one ITN for every two household members) for satisfactory protection and the epidemiological impact of ITNs as a vector control tool was nearly met in Koksora CHC.

After 43 months of field use, >50% of ITNs in foothill villages and 50% (45.9%) of ITNs in plain villages had holes. According to the current study, 15.8%, 59.1% and 25.1% of ITNs were determined to be in good, serviceable and replacement conditions, correspondingly. Mixing good and serviceable conditions, more than 70% of the ITNs in the current investigation had a useful life of more than 3 years (after 43 months of field use). According to reports, ITNs can prevent blood feeding at the household level even if they are damaged (65 < pHI < 642). Pyrethroids impregnated or coated on the net have a repellent effect, which contributes in part to this [34]. However, a mosquito may easily penetrate an ITN if its pHI value is high (>643) in order to feed on a sleeping human before resting outside the ITNs [35].

Taking into account both attrition and physical durability, >90% of ITNs distributed in Koksara CHC, Kalahandi district, Odisha, survived after 43 months. In addition to being sold, stolen or given away, wear and tear was the leading cause of attrition. In contrast, very few ITNs were observed being utilised for alternative reasons. After 30 months, over 50% of the ITNs (PermaNet 2.0) in Zambia were still operational [33].

According to the physical integrity of the ITNs, 15.8% and 59.1% of the nets were in good and usable condition, respectively. If these nets were combined, the user might still benefit from getting protection from the bites of vector mosquito, even after the expected life span of 3 years of ITNs. This demonstrates the community's social approval of the ITNs and their awareness of their promotion as a vector control measure to reduce the prevalence of malaria in the area. 25% of the ITNs were placed in the ‘needs replacement’ category after 43 months because they were regarded as being of little to no use to the user.

Chemical content analyses revealed a decrease in insecticidal content with a mean deltamethrin residue of 15.3 mg/m^2^, representing a loss of 72.2% compared with the insecticidal content of 55 mg/m^2^ in the fresh nets.

61.6% of the households in the current survey had maintained their end‐of‐life ITNs secure. In 7.3% of households in Koksara CHC, it was found that they had reused ITNs that had reached the end of life. These end‐of‐life ITNs were repurposed for a variety of indoor and outdoor uses, including covering earthen pots and small mud pots used for toddy tapping, protecting newly hatched chickens from predators, fencing, protecting plant saplings in nurseries, fishing and other purposes (13.7%). The remaining 17.4% of households had discarded the ITNs. The members of the household are neither protected nor injured by these recycled nets (Roll Back Malaria Partnership…consensus statement on repurposing). These neutral nets were repurposed to aid in protecting the limited resources of the underprivileged communities. Repurposing has been viewed as a means of safeguarding resources in Kenya [34]. It has been reported that old nets used as screens, curtains or ceiling covers are more effective at protecting against mosquito bites than households that dispose of end‐of‐life ITNs or neutrally repurpose them elsewhere [34, 35, 36]. In 17.4% of the households, we found that end‐of‐life ITNs had been discarded. In addition to the pollution brought on by the nets' ripped or decomposing plastic components, concerns have been expressed regarding the potential public health and environmental risk posed by the end‐of‐life unused nets that may still contain trace amounts of pyrethroid pesticide content (WHO, 2014).

In order to devise a disposal strategy for ITNs that have reached the end of their useful lives, this study assessed the knowledge and perspectives of key stakeholders, including those involved and not involved in the control of VBDs, on malaria and ITNs. The results indicate broad consensus regarding the transmission of diseases by mosquitoes and the breeding grounds for mosquitoes in water bodies. According to the professionals, the number of malaria cases has decreased in comparison with the previous years. All of these professionals agreed that IRS and ITNs were the most effective vector control tools for managing malaria.

It's interesting that the professional knew how long an ITN could last. The advice or recommendations of the professionals about the return of the end‐of‐life ITNs to the Government and their concern for safe disposal are also crucial.

## CONCLUSIONS AND THE WAY FORWARD

In the Koksara CHC, misuse of bed net was not observed, but we did notice net repurposing. The end‐of‐life ITNs were repurposed for fencing, covering saplings in nurseries, covering clay pots, small mud pots and protecting chickens from predators.

Community opinion regarding the disposal of end‐of‐life ITNs has been incorporated into the development of evidence‐based decision‐support data. This data will facilitate the formulation of a plan for the systematic collection and secure, cost‐effective disposal of used nets. Currently, the National Program (National Centre for Vector Borne Diseases Control (NCVBDC), Directorate General of Health Services, Ministry of Health & Family Welfare, GOI) lacks an operationally viable strategy for the safe disposal of end‐of‐life ITNs. To establish comprehensive guidelines for their collection and disposal, a thorough analysis is required, considering the challenges associated with ITN disposal at both programmatic and household levels.

This study can serve as a foundation for developing sustainable, long‐term and cost‐effective solutions for the disposal of end‐of‐life ITNs. Once this strategy is demonstrated to be operationally feasible, a national policy for the end‐of‐life ITN disposal can be formulated. Such evidence is essential for establishing comprehensive national guidelines, which are currently lacking. We recommend the development of practical, durable and financially viable methods for the disposal of end‐of‐life ITNs. The authors propose the following actions: (1) Establish a district‐level framework for data collection, transportation and storing (warehousing). (2) Explore potential disposal methods in collaboration with the fabric recycling industry and (3) Conduct a cost‐effectiveness analysis of secure destruction methods for end‐of‐life ITNs.

## FUNDING INFORMATION

This study was approved by the Director General and supported by the Indian Council of Medical Research (ICMR), New Delhi, India.

## CONFLICT OF INTEREST STATEMENT

The authors declare that they have no competing interests.

## Data Availability

Relevant data obtained from the CHCs are available within the manuscript in the form of primary tables and graphs. Data are available on reasonable request to ICMR‐VCRC.

## References

[1] Lengeler C . Insecticide‐treated bed nets and curtains for preventing malaria. Cochrane Database Syst Rev. 2004;2:CD000363. 10.1002/14651858.cd000363.pub2 15106149

[2] Van Remoortel H , De Buck E , Singhal M , Vandekerckhove P , Agarwal SP . Effectiveness of insecticide‐treated and untreated nets to prevent malaria in India. Trop Med Int Health. 2015;20(8):972–982. 25877758 10.1111/tmi.12522

[3] WHO . World malaria report. Geneva: World Health Organization; 2023.

[4] Zaim M , Aitio A , Nakashima N . Safety of pyrethroid‐treated mosquito nets. Med Vet Entomol. 2000;14(1):1–5. 10.1046/j.1365-2915.2000.00211.x 10759305

[5] WHOPES 13th Working Group Meeting . World Health Organization. Geneva: World Health Organization. Ref: WHO/HTM/NTD/WHOPES/2009.5 10 April 2016.

[6] Hakizimana E , Cyubahiro B , Rukundo A , Kanayiza A , Mutabazi A Beach R , et al. Monitoring long‐lasting insecticidal net (ITNS) durability to validate net serviceable life assumptions, in Rwanda. Malar J. 2014; 13:344. 25174414 10.1186/1475-2875-13-344 PMC4161833

[7] Minakawa N , Dida GO , Sonye GO , Futami K , Kaneko S . Unforeseen misuses of bed nets in fishing villages along Lake Victoria. Malar J. 2008;7:165. 10.1186/1475-2875-7-165 18752662 PMC2532690

[8] Efong U . Fighting Malaria is Going to Take More than Just Nets. 2015 [cited 2019 Jan 29]. Available from: https://medicalexpress.com/news/2015-02-malaria-nets.html

[9] Shah S . In Africa anti‐malaria nets go unused by recipients. *Los Angeles Times*. 2 May 2010 [cited 2019 Jan 29]. Available from: https://www.latimes.com/archives/la-xpm-2010-may-02-la-oe-shah-20100502.-story.html

[10] Utuk NM , Abasiattai AM , Ugege WE . Misuse of long‐lasting insecticidal nets in Akwa Ibom state, South‐South Nigeria. J Case Rep Images Med. 2017;3:30–32.

[11] Roll Back Malaria Partnership . Consensus statement on repurposing ITNs: applications for BCC messaging and actions at the country level. 2018 [cited 2019 Jun 30]. Available from: https://www.vector-works.org/wp-content/uploads/ConsensusStatemenRepurposing-of-ITNs-2018-11-28.pdf Accessed 30 June 2020.

[12] Eisele TP , Thwing J , Keating J . Claims about the misuse of insecticide‐treated mosquito nets: are these evidence‐based? PLoS Med. 2011;8(4):e1001019. 10.1371/journal.pmed.1001019 21532734 PMC3075220

[13] Bennett A , Smith SJ , Yambasu S , Jambai A , Alemu W , Kabano A , et al. Household possession and use of insecticide‐treated mosquito nets in Sierra Leone 6 months after a national mass‐distribution campaign. PLoS One. 2012;7(5):e37927. 10.1371/journal.pone.0037927 22666414 PMC3362537

[14] Koenker H , Kilian A , Zegers de Beyl C , Onyefunafoa EO , Selby RA , Abeku T , et al. What happens to lost nets: a multi‐country analysis of reasons for LLIN attrition using 14 household surveys in four countries. Malar J. 2014;13:464. 10.1186/1475-2875-13-464 25430956 PMC4258934

[15] Doda Z , Solomon T , Loha E , Gari T , Lindtjørn B . A qualitative study of use of long‐lasting insecticidal nets (LLINs) for intended and unintended purposes in Adami Tullu, East Shewa Zone, Ethiopia. Malar J. 2018;17(1):69. 10.1186/s12936-018-2209-5 29409511 PMC5801687

[16] National framework for malaria elimination in India (2016–2030). New Delhi: Directorate of National Vector Borne Disease Control Programme (NVBDCP), Directorate General of Health Services (DGHS), Ministry of Health & Family Welfare Government of India; 2016.

[17] National strategic plan (NSP) malaria elimination in India (2017–b2022). New Delhi: Directorate of National Vector Borne Disease Control Programme (NVBDCP), Directorate General of Health Services (DGHS) Ministry of Health & Family Welfare Government of India; 2017.

[18] Pradhan MM , Meherda PK . Malaria elimination drive in Odisha: hope for halting the transmission. J Vector Borne Dis. 2019;56(1):53–55. 10.4103/0972-9062.257775 31070166

[19] World Health Organization . Guidelines WHO. For laboratory and field‐testing of long‐lasting insecticidal nets. Geneva: World Health Organization; 2013.

[20] Yewhalaw D , Asale A , Tushune K , Getachew Y , Duchateau L , Speybroeck N . Bio‐efficacy of selected long‐lasting insecticidal nets against pyrethroid resistant *Anopheles arabiensis* from South‐Western Ethiopia. Parasit Vectors. 2012;5:159. 10.1186/1756-3305-5-159 22871143 PMC3485103

[21] Kilian A , Byamukama W , Pigeon O , Atieli F , Duchon S , Phan C . Long‐term field performance of a polyester‐based long‐lasting insecticidal mosquito net in rural Uganda. Malar J. 2008;7:49. 10.1186/1475-2875-7-49 18355408 PMC2330059

[22] Fettene M , Balkew M , Gimblet C . Utilization, retention and bio‐efficacy studies of PermaNet in selected villages in Buie and Fentalie districts of Ethiopia. Malar J. 2009;8:114. 10.1186/1475-2875-8-114 19480712 PMC2694207

[23] Van Roey K , Sovannaroth S , Sochantha T , Srey Touch MS , Pigeon O , Sluydts V , et al. A phase III trial to evaluate the efficacy, fabric integrity and community acceptance of Netprotect® using a recommended long‐lasting insecticidal net as positive control. Malar J. 2016;13:256. 10.1186/1475-2875-13-256 PMC4105388 24998677

[24] Sahu SS , Keshaowar AV , Thankachy S , Panigrahi DK , Acharya P , Balakrishnan V , et al. Evaluation of bio‐efficacy and durability of long‐lasting insecticidal nets distributed by malaria elimination programme in Eastern India. Malar J. 2020;19(1):186. 10.1186/s12936-020-03260-2 32448316 PMC7247230

[25] Sahu SS , Gunasekaran K , Krishnamoorthy N , Vanamail P , Mathivanan A , Manonmani A , et al. Bionomics of *Anopheles fluviatilis* and *Anopheles culicifacies* (Diptera: Culicidae) in relation to malaria transmission in East‐Central India. J Med Entomol. 2017;54(4):821–830. 10.1093/jme/tjx065 28399290 PMC5850663

[26] Boyer S , Pothin E , Randriamaherijaona S , Rogier C , Kesteman T . Testing bio‐efficacy of insecticide‐treated nets with fewer mosquitoes for enhanced malaria control. Sci Rep. 2018;8(1):16769. 10.1038/s41598-018-34979-3 30425283 PMC6233220

[27] Ahogni IB , Salako AS , Akinro B , Sovi A , Gnanguenon V , Azondekon R , et al. Physical integrity and survivorship of long‐lasting insecticidal nets distributed to households of the same socio‐cultural community in Benin, West Africa. Malar J. 2020;19(1):58. 10.1186/s12936-020-3138-7 32019586 PMC7001382

[28] Randriamaherijaona S , Raharinjatovo J , Boyer S . Durability monitoring of long‐lasting insecticidal (mosquito) nets (LLINs) in Madagascar: physical integrity and insecticidal activity. Parasit Vectors. 2017;10(1):564. 10.1186/s13071-017-2419-7 29132421 PMC5683549

[29] National strategic plan for malaria elimination in India (2017–22). New Delhi: National Vector Borne Disease Control Programme; 2017.

[30] Division of Malaria Control [Ministry of Public Health and Sanitation] , Kenya National Bureau of Statistics , ICF Macro . 2010 Kenya malaria indicator survey. Nairobi: DOMC, KNBS and ICF Macro; 2011.

[31] Kilian A , Byamukama W , Pigeon O , Gimnig J , Atieli F , Koekemoer L , et al. Evidence for a useful life of more than three years for a polyester‐based long‐lasting insecticidal mosquito net in Western Uganda. Malar J. 2011;10:299. 10.1186/1475-2875-10-299 21992483 PMC3212829

[32] Azondekon R , Gnanguenon V , Oke‐Agbo F , Houevoessa S , Green M , Akogbeto M . A tracking tool for long‐lasting insecticidal (mosquito) net intervention following a 2011 national distribution in Benin. Parasit Vectors. 2014;7:6. 10.1186/1756-3305-7-6 24387635 PMC3892047

[33] Tan KR , Coleman J , Smith B , Hamainza B , Katebe‐Sakala C , Kean C , et al. A longitudinal study of the durability of long‐lasting insecticidal nets in Zambia. Malar J. 2016;15:106. 10.1186/s12936-016-1154-4 26891696 PMC4759777

[34] Santos EM , Coalson JE , Munga S , Agawo M , Jacobs ET , Klimentidis YC , et al. “After those nets are torn, most people use them for other purposes”: an examination of alternative bed net use in western Kenya. Malar J. 2020;19(1):272. 10.1186/s12936-020-03342-1 32727452 PMC7390200

[35] Baume CA , Reithinger R , Woldehanna S . Factors associated with use and non‐use of mosquito nets owned in Oromia and Amhara regional states, Ethiopia. Malar J. 2009;8:264. 10.1186/1475-2875-8-264 19930654 PMC2796673

[36] Loll DK , Berthe S , Faye SL , Wone I , Koenker H , Arnold B , et al. User‐determined end of net life in Senegal: a qualitative assessment of decision‐making related to the retirement of expired nets. Malar J. 2013;12:337. 10.1186/1475-2875-12-337 24053789 PMC3856457

